# Supplementary material for: Comparative multiomics analysis of cell physiological state after culture in a basket bioreactor
Source: Sci Rep. 2022 Nov 23;12:20161. doi: 10.1038/s41598-022-24687-4 (PMC9686226; doi:10.1038/s41598-022-24687-4)

66037.1:193883344:193883421:-@NW_023666037.1:193883004:193883048:-@NW_023666037.1:193882099:193

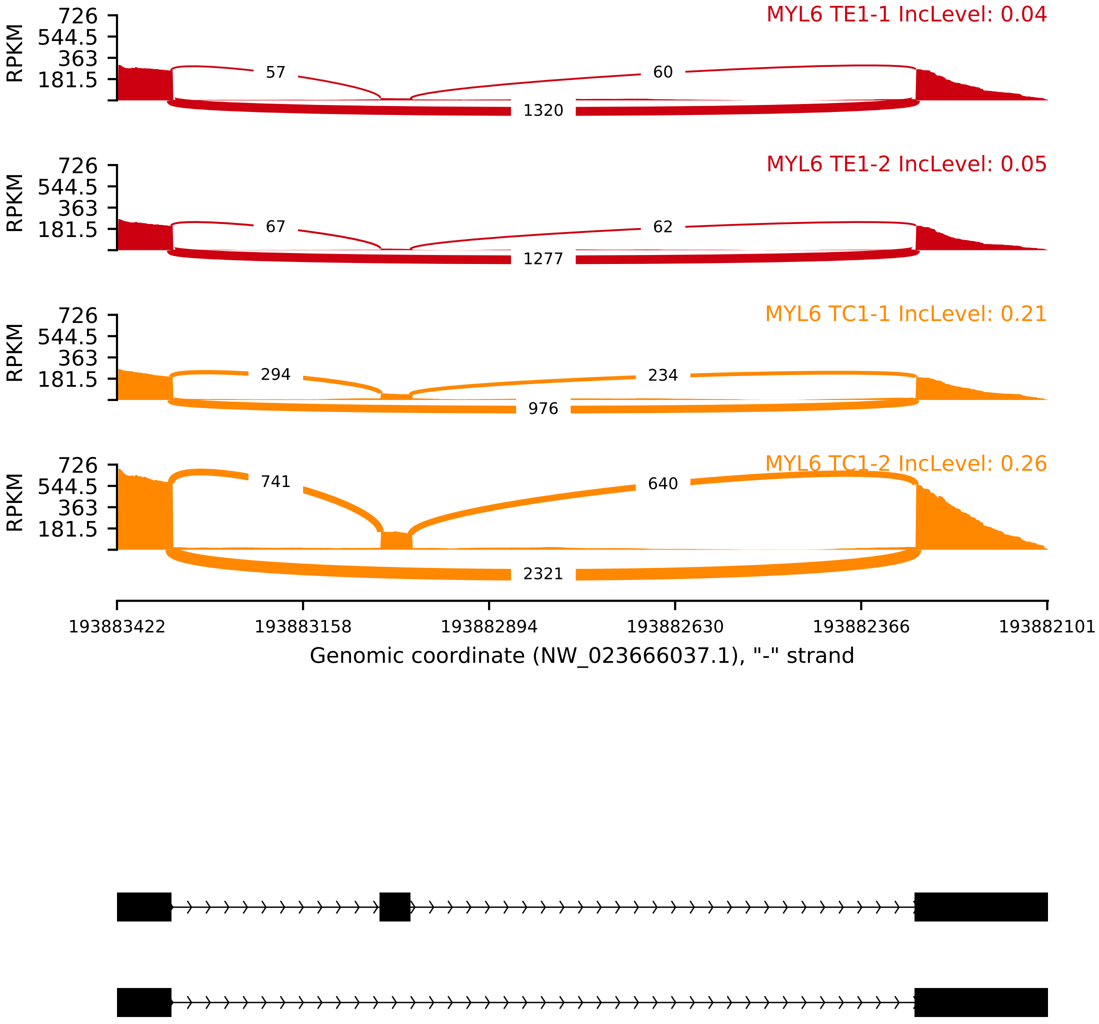

Supplement: Supplementary file 1 — Supplementary Information 1. [file 41598_2022_24687_MOESM1_ESM.zip › raw data/Transcriptomic raw data/7.AS/2.dasplot/TE1vsTC1/SE/6_MYL6_NW_023666037.1_193883344_193883421_-@NW_023666037.1_193883004_193883048_-@NW_023666037.1_193882099_193882288_-.pdf]
